# Sperm DNA methylation mediates the association of male age on reproductive outcomes among couples undergoing infertility treatment

**DOI:** 10.1038/s41598-020-80857-2

**Published:** 2021-02-05

**Authors:** Oladele A. Oluwayiose, Haotian Wu, Hachem Saddiki, Brian W. Whitcomb, Laura B. Balzer, Nicole Brandon, Alexander Suvorov, Rahil Tayyab, Cynthia K. Sites, Lisa Hill, Chelsea Marcho, J. Richard Pilsner

**Affiliations:** 1 Department of Environmental Health Sciences, School of Public Health and Health Sciences, University of Massachusetts Amherst, 173A Goessmann, 686 North Pleasant Street, Amherst, MA 01003 USA; 2 Department of Biostatistics and Epidemiology, School of Public Health and Health Sciences, University of Massachusetts Amherst, 715 North Pleasant Street, Amherst, MA USA; 3 Division of Reproductive Endocrinology and Infertility, Baystate Medical Center, 759 Chestnut Street, Springfield, MA USA; 4 Present Address: Department of Environmental Health Sciences, Columbia University Mailman School of Public Health, 722 W 168th St, New York, NY 10032 USA

**Keywords:** Epigenetics, Ageing

## Abstract

Parental age at time of offspring conception is increasing in developed countries. Advanced male age is associated with decreased reproductive success and increased risk of adverse neurodevelopmental outcomes in offspring. Mechanisms for these male age effects remain unclear, but changes in sperm DNA methylation over time is one potential explanation. We assessed genome-wide methylation of sperm DNA from 47 semen samples collected from male participants of couples seeking infertility treatment. We report that higher male age was associated with lower likelihood of fertilization and live birth, and poor embryo development (p < 0.05). Furthermore, our multivariable linear models showed male age was associated with alterations in sperm methylation at 1698 CpGs and 1146 regions (q < 0.05), which were associated with > 750 genes enriched in embryonic development, behavior and neurodevelopment among others. High dimensional mediation analyses identified four genes (*DEFB126, TPI1P3, PLCH2* and *DLGAP2)* with age-related sperm differential methylation that accounted for 64% (95% CI 0.42–0.86%; p < 0.05) of the effect of male age on lower fertilization rate. Our findings from this modest IVF population provide evidence for sperm methylation as a mechanism of age-induced poor reproductive outcomes and identifies possible candidate genes for mediating these effects.

## Introduction

Over the past several decades, a trend toward delayed childbirth has led to increases in parental age at the time of conception, especially in developed countries^1^. This delayed parenthood is attributed to secular and socioeconomic factors^2^, reproductive technological advancement^3^, and higher levels of post-graduate education^4^. To date, emphasis has been on the effects of advanced maternal age on adverse reproductive and offspring health^5–7^. However, new evidence suggests that, irrespective of maternal age, higher male age contributes to longer time-to-conception^8^, poor pregnancy outcomes^8^, lower odds of live birth in infertile cohort^9^, and adverse health of the offspring at later life^10–12^. For instance, higher male age is associated with poor outcomes in offspring, including: reduced life span in mice^13^; increased susceptibility to early development of cancer^14^; chromosomal abnormalities^15^; and neurodevelopmental disorders such as schizophrenia^16^ and autism^11,12,17,18^.

Despite the host of adverse reproductive health outcomes associated with higher male age at conception, the precise mechanism remains unclear. Proposed mechanisms include reduced sperm quality^19^, poor sperm DNA integrity^20^, the accumulation of de novo mutations from the self-renewal and differentiation of spermatogonial stem cells^21^, and adverse psychosocial environment^22^. Age-associated adverse reproductive outcomes, however, are likely multifactorial, and as such, additional biological pathways are important to identify to inform avenues of translational research to improve reproductive health.

**Figure 1:**
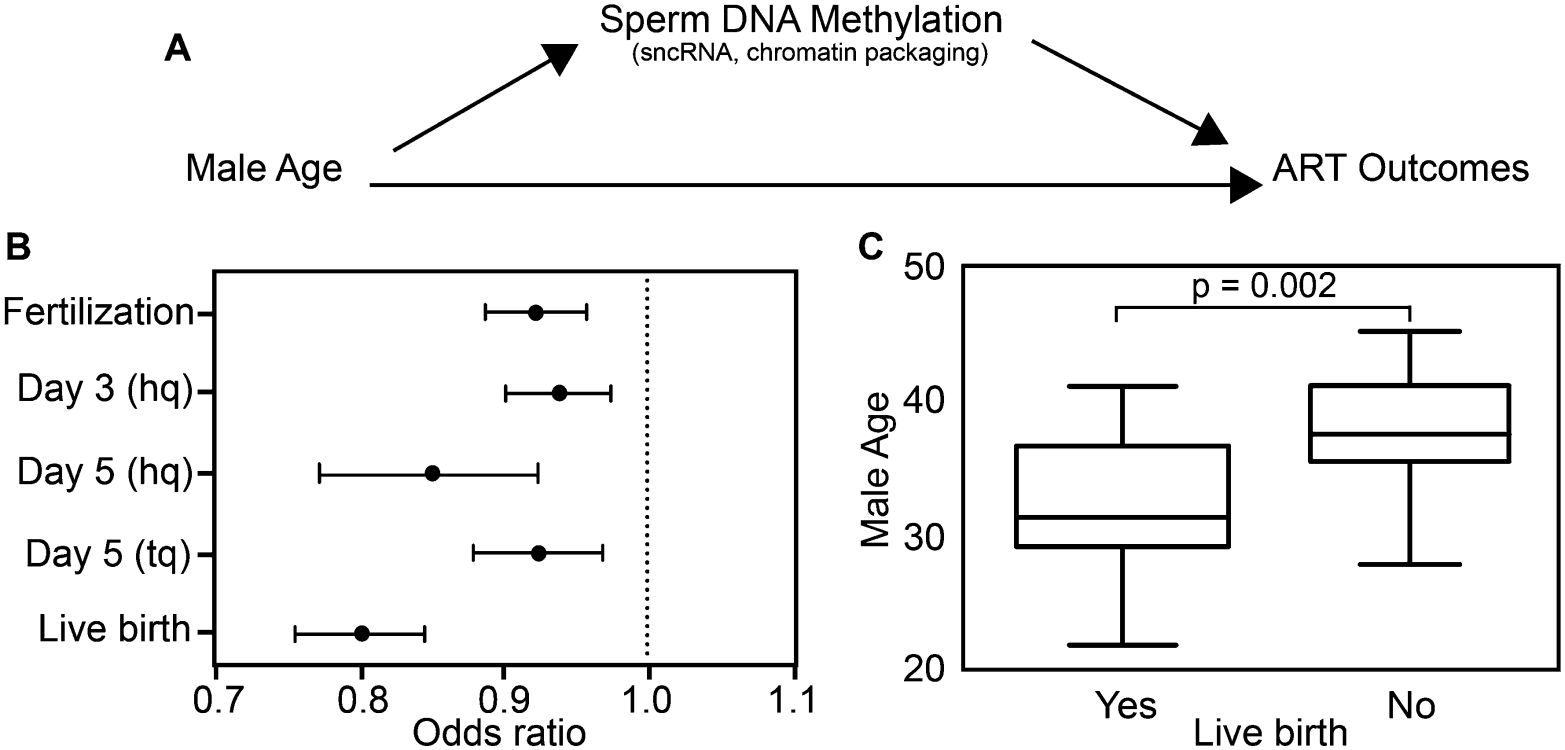
Male age and assisted reproductive technology (ART) outcomes. (**A**) Flow chart of our a priori hypothesis that sperm DNA methylation mediates the effect of male age on ART outcomes. Other potential epigenetic mechanisms not included in this study are listed below sperm DNA methylation including small noncoding RNA and chromatin packaging. (**B**) Odds ratios between male age and ART outcomes adjusting for male BMI, infertility, smoking and female age. (**C**) Unadjusted box plots of male age for couples with and without successful live births (t-test: − 3.48 p-value = 0.002). hq: high quality embryo; tq: transfer quality embryo.

Age-associated changes in sperm epigenetics is another potential mechanism that may explain the association of higher male age with adverse reproductive outcomes, but limited data exist evaluating this hypothesis^23^. The epigenetic landscape of cells is determined by the interaction of DNA methylation, chromatin structure governed by histone modifications, and non-coding RNAs. Of particular relevance are the recent findings in mice and human indicating that the sperm epigenetic landscape in adult males can be modified by environmental conditions such as low protein diet^24^, cannabis use^25^, physical activity^26^, stress^27^ and environmental chemicals such as phthalates^28^. Several studies have reported age associated changes in DNA methylation in somatic tissues^29–31^ with recent advances enabling prediction of adverse health outcomes including lifespan^32–34^. However, the impact of male age on methylation profiles of male germ cells is limited^35,36^ and most recently, it has been shown that age-associated sperm methylation may not persist across generations^37^. However, to date, no study has examined age-associated sperm methylation profiles with respect to reproductive outcomes. Research in clinical populations of couples seeking treatment for infertility allows direct observation of reproductive events including fertilization, embryo development, and pregnancy outcomes. However, such observations are largely occult among the general population. Hence, the objective of this study was to examine the role of sperm DNA methylation as a potential mechanism by which male age affects assisted reproductive technology (ART) outcomes among couples seeking infertility treatment (Fig. 1A).

## Results

### Male age and assisted reproductive outcomes

Outcomes of ART treatment considered in these analyses include fertilization of retrieved oocytes; high quality embryo status at multiple time points; and whether the treatment resulted in a live birth. Characteristics of the couple participants (n = 47) are described in Table 1. Male age ranged from 21 to 45 years with the majority of the participants (53.2%) between 30 and 40 years of age. Similarly, female partners’ age ranged from 24 to 42 years and were mostly (74%) between 30 and 40 years of age. In multivariable logistic regression models adjusting for male BMI, male infertility, male smoking and female age, each 1 year higher male age was associated with lower likelihood of fertilization (Fig. 1B. OR = 0.92, 95% confidence interval (CI) 0.89, 0.96); day 3 high quality embryos (OR = 0.94; 95% CI 0.90, 0.98), day 5 high quality embryos (OR = 0.85; 95% CI 0.77, 0.93), and day 5 transfer quality embryos (OR = 0.93; 95% CI 0.88, 0.97). Additionally, each 1 year higher male age was associated with lower likelihood of live birth (OR = 0.80; 95% CI 0.76, 0.85); likewise average male age was higher among couples without successful live birth compared to those with successful live birth (Fig. 1C. 35.1 ± 4.0 and 32.4 ± 4.4 years, respectively; p < 0.05). As a sensitivity analysis, models were also run stratified on infertility status to evaluate whether observed male age associations differed between fertile and infertile strata. Comparable estimates were observed, and results did not appreciably change the overall interpretation of our findings (Supplementary Table S1).

**Table 1:** Characteristics of SEEDS participants (n = 47).

Individual characteristics	Male (n (%))	Female (n (%))
**Age (years)**		
< 26	2 (4.2)	1 (2.1)
26–30	7 (14.9)	8 (17.0)
31–35	8 (17.0)	16 (34.0)
36–40	17 (36.2)	19 (40.4)
> 40	13 (27.7)	3 (6.4)
**BMI (kg/m**^**2**^**)**^**a**^		
< 25	10 (21.3)	17 (36.2)
25–30	19 (40.4)	11 (23.4)
30+	17 (36.2)	19 (40.4)
**Race**^**b**^		
White (non-hispanic)	36 (76.6)	44 (93.6)
Others	4 (8.5)	2 (4.3)
**Current smoking**^**c**^		
Yes	4 (8.5)	0 (0%)
No	43 (91.5)	47 (100%)
**Diagnosed infertility**		
Male factor only	5 (10.6)	
Female factor only	20 (42.5)	
Both	6 (12.7)	
Unidentifiable	16 (34.0)	

Semen parameters	Median	n (%) < WHO reference
Normal morphology (%)	5.5	16 (34.0)
Total motility (%)	58	8 (17.0)
Sperm count (10^6^)	91	2 (4.3)
Semen volume (mL)	3	9 (19.2)
Sperm concentration (10^6^/mL)	47.5	12 (25.5)

Cycle-specific characteristics	(Mean ± SD or n (%))	
Oocyte retrieved	15.1 ± 8.0	
Fertilized embryos	9.0 ± 6.6	
**High quality embryos**		
Day 3	5.1 ± 4.1	
Day 5	0.9 ± 1.4	
Live birth	15 (31.9)	

^a^Missing male BMI data (n = 1).

^b^Missing race data (Male, n = 7 and female, n = 1).

^c^Males based on urinary cotinine and females based on self-report.

### Male age and sperm methylation

**Figure 2:**
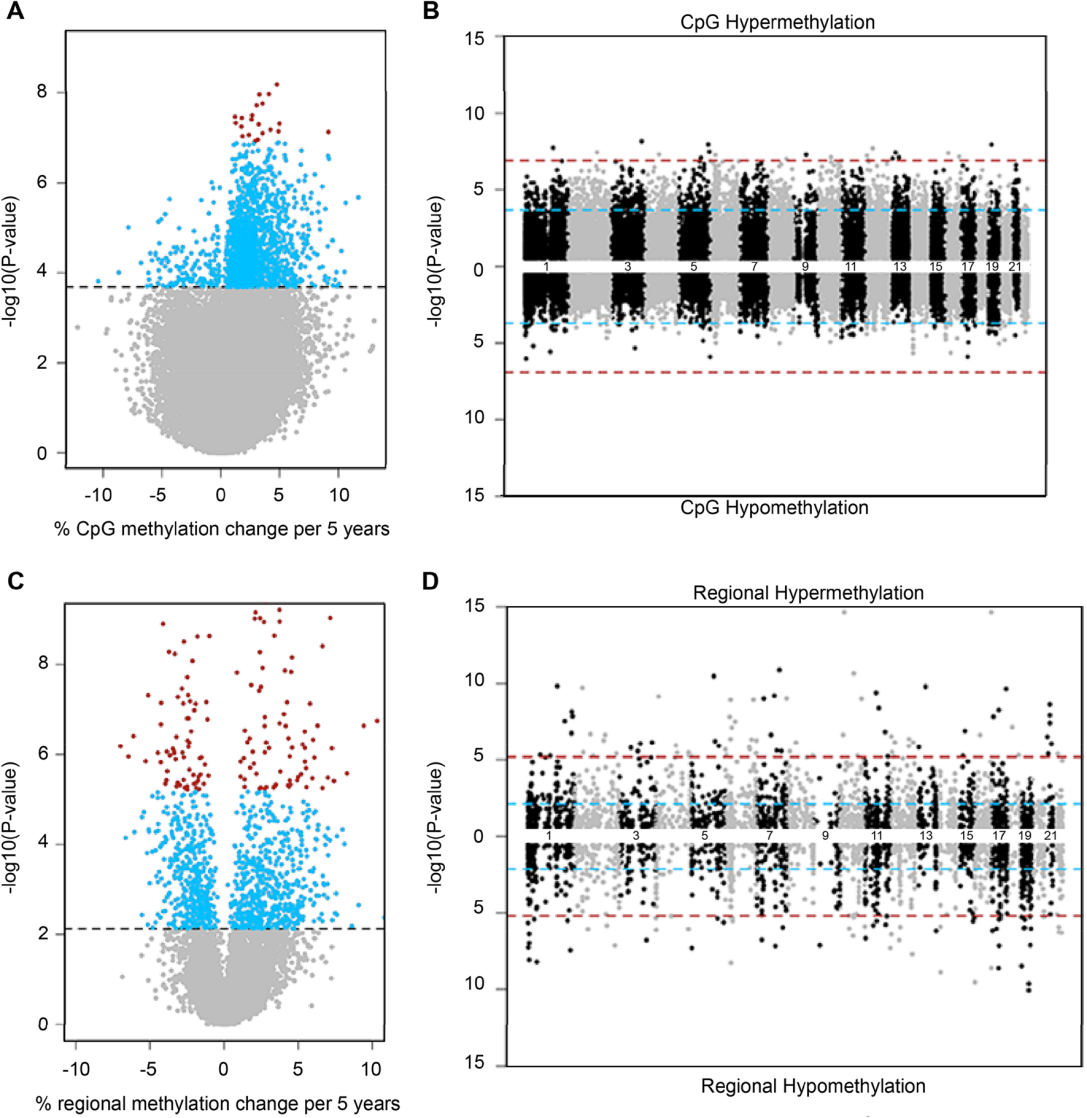
Volcano and Miami plots of age-associated sperm methylation at individual CpGs and regionally. Volcano plots show methylation change per 5 years of age adjusting for male BMI, infertility and smoking at (**A**) individual CpGs and (**C**) regionally where grey, blue and red-colored data points representing non-significant, FDR significant, and Bonferroni significant, respectively. Miami plots show the chromosomal distribution of age-associated sperm methylation at (**B**) individual CpGs and (**D**) regionally where blue and red dashed lines represent FDR and Bonferroni cut-off levels, respectively.

In order to evaluate the extent to which the observed associations of higher male age with poorer ART outcomes might be mediated by sperm epigenetics, we next considered relations of male age with sperm DNA methylation of individual CpG sites and regionally. After adjusting for male BMI, male infertility and male smoking, male age was associated with 1698 individual sperm CpG sites (q < 0.05). The majority of CpGs (91%; n = 1546) showed an increase in methylation with respect to male age, whereby for every 5-year increase in male age, an increase in methylation at these CpGs ranged from 0.2 to 11.7% (mean ± SD: 2.8% ± 1.8%). Of the CpG sites associated with male age, 19 CpGs remained significant after Bonferroni correction (Fig. 2A,B; Supplementary Table S2).

Analysis was also conducted to identify differentially methylated regions (DMRs) associated with male age. In multivariable linear regression analyses adjusted for covariates as described previously, male age was associated with 1146 DMRs (q < 0.05), of which roughly 10% (n = 156) were statistically significant at the Bonferroni correction level (Fig. 2C,D; Supplementary Table S3). Unlike the overwhelming hypermethylation observed with individual CpGs, age-associated sperm DMRs represented a mix of hypomethylation (43%) and hypermethylation (57%) regions; however, the differences in percent methylation of DMRs per 5 years of age were similar to those of individual CpGs.

### Genomic features of age-associated sperm DMRs

**Figure 3:**
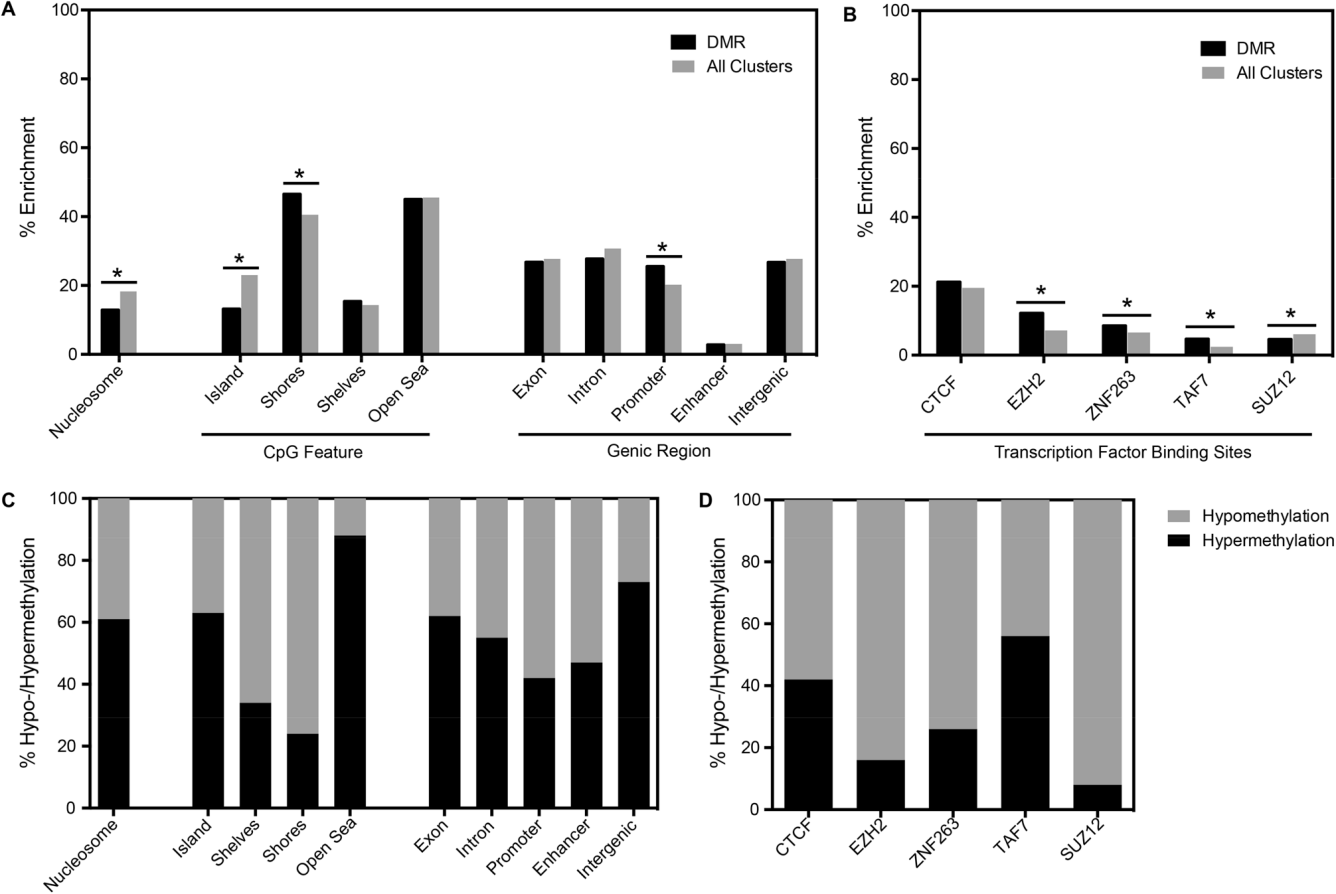
Localization of age-associated sperm DMRs. Enrichment analysis of all methylation clusters compared to DMRs at (**A**) nucleosome, CpG feature, and genic regions and (**B**) transcription factor binding sites and (**C**,**D**) their respective age-associated methylation patterns (**C**,**D**). Categories not exclusive, numbers may not add up to 100%.

To better understand the localization of the age-associated sperm DMRs, we next examined DMRs based on their genomic features. Age-associated sperm DMRs were depleted in regions previously identified with retained nucleosomes reported by Donkin et al.^38^ compared to all clusters (Fig. 3A: 12.9% vs. 18.3%; p = 4.7 × 10^–6^). For CpG features, compared to all clusters, age-associated DMRs were enriched in CpG shores (45.5% vs. 40.5%, p < 0.002) and depleted in CpG islands (13.2% vs. 23.0%, p = 2.8 × 10^–15^). For genic regions, age-associated DMRs were enriched in promoter regions compared to all clusters (Fig. 3A: 25.5% vs. 20.2%, p = 5.0 × 10^–5^). Of the 148 transcription factors binding sites examined, three were enriched (*EZH2*, *ZNF263*, and *TAF7*) while *SUZ12* was depleted for age-associated DMRs compared to all clusters (Fig. 3B and Supplementary Table S4). Finally, the loss or gain of methylation in sperm DMRs was approximately equally distributed (40–60%) in most genomic features. However, pronounced hypermethylation of sperm DMRs was observed in open sea (88%) and intergenic (73%) regions, whereas shelves (66%) and shores (76%) were predominately hypomethylated (Fig. 3C). Hypomethylation of sperm DMRs was also pronounced among transcription factor bindings sites of *EZH2* (84%), *ZNF263* (74%) and *SUZ12* (92%) (Fig. 3D).

### Pathway analyses of age-associated sperm DMRs

**Figure 4:**
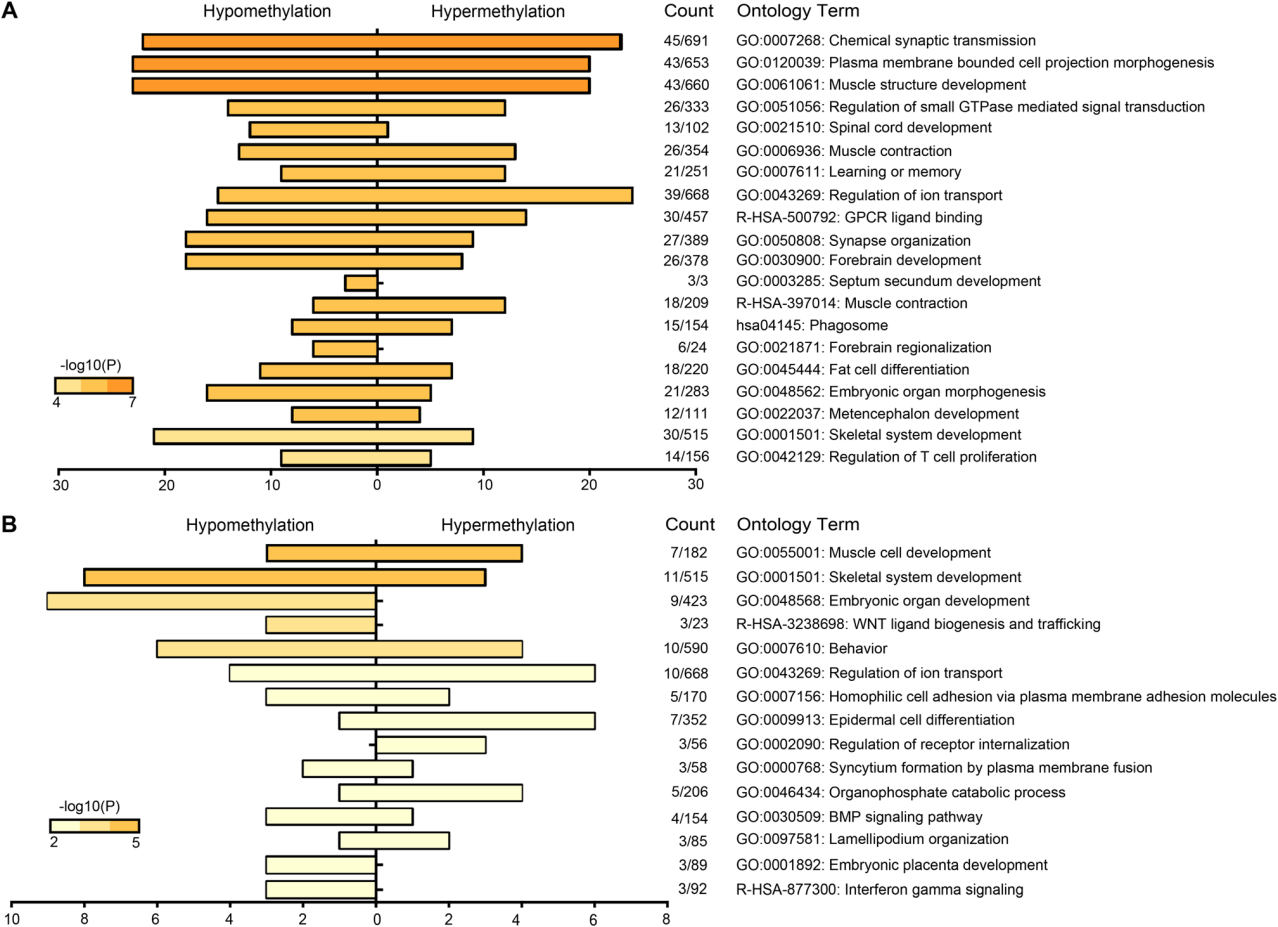
Pathway analysis of age-associated sperm DMRs. (**A**) FDR-adjusted DMRs (n = 1146) and (**B**) Bonferroni-adjusted DMRs (n = 156).

To determine the potential biological significance of age-associated sperm DMRs, we conducted pathway analyses by restricting analyses to DMRs located within 1500 bp distance from TSS of genes. Of the 1146 sperm DMRs identified, 783 unique genes remained for pathway analysis (Supplementary Table S5). Top enriched pathways were largely those involving early-life development, such as muscle structure development and embryonic organ morphogenesis, as well as neurodevelopmental pathways including spinal cord and forebrain development, neuron differentiation and regionalization, and behavior (Fig. 4A). Likewise, when we restricted our analyses to the 101 unique genes associated with the 156 statistically significant sperm DMRs at the Bonferroni correction level, behavior and developmental pathways were highly enriched including homophilic cell adhesion, epithelial cell differentiation, *WNT* ligand biogenesis and trafficking, and embryonic placenta development (Fig. 4B and Supplementary Table S6).

**Figure 5:**
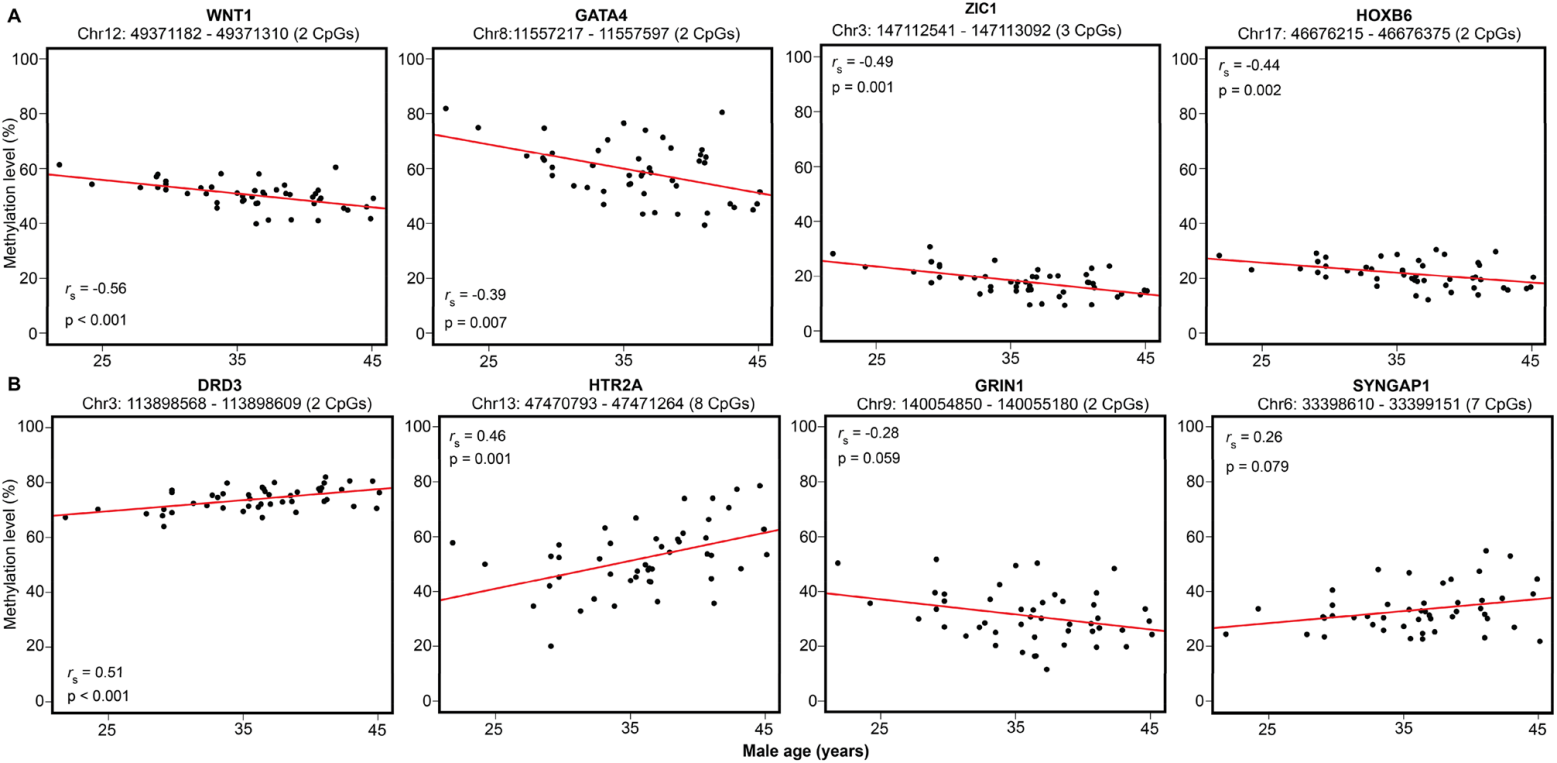
Scatterplots of age-related sperm DNA methylation. Selected genes associated with (**A**) early embryonic development (**B**) behavioral patterns. Genomic locations based on GRCh37/hg19 2009 human assembly.

Scatter plots depict the associations between male age and sperm DNA methylation of a subset of genes important for early embryonic development and behavior (Fig. 5). Male age was associated with a loss of sperm methylation at loci of key development genes (Fig. 5A) such as of wingless/integrated 1 (*WNT1*; *rho* = − 0.56, p < 0.001), GATA binding protein 4 (*GATA4*; *rho* = − 0.45, p = 0.007), zinc finger of the cerebellum 1 (*ZIC1*; *rho* = − 0.49, p = 0.001) and homeobox B6 (*HOXB6*; *rho* = − 0.44, p = 0.002). For behavior-associated genes, male age was significantly associated with increases in sperm methylation in dopamine receptor D3 (*DRD3*; *rho* = 0.51, p < 0.001), and 5-hydroxytryptamine receptor 2A (*HTR2A*; *rho* = 0.46, p = 0.001). Altered methylation was also observed in glutamate ionotropic receptor NMDA type subunit 1 (*GRIN1*; *rho* = − 0.28, p = 0.059) and synaptic Ras GTPase activating protein 1 (*SNYGAP1*; *rho* = 0.26, p = 0.079), although borderline significant with spearman correlations (Fig. 5B).

### Age-associated sperm methylome profiles and ART outcomes

**Figure 6:**
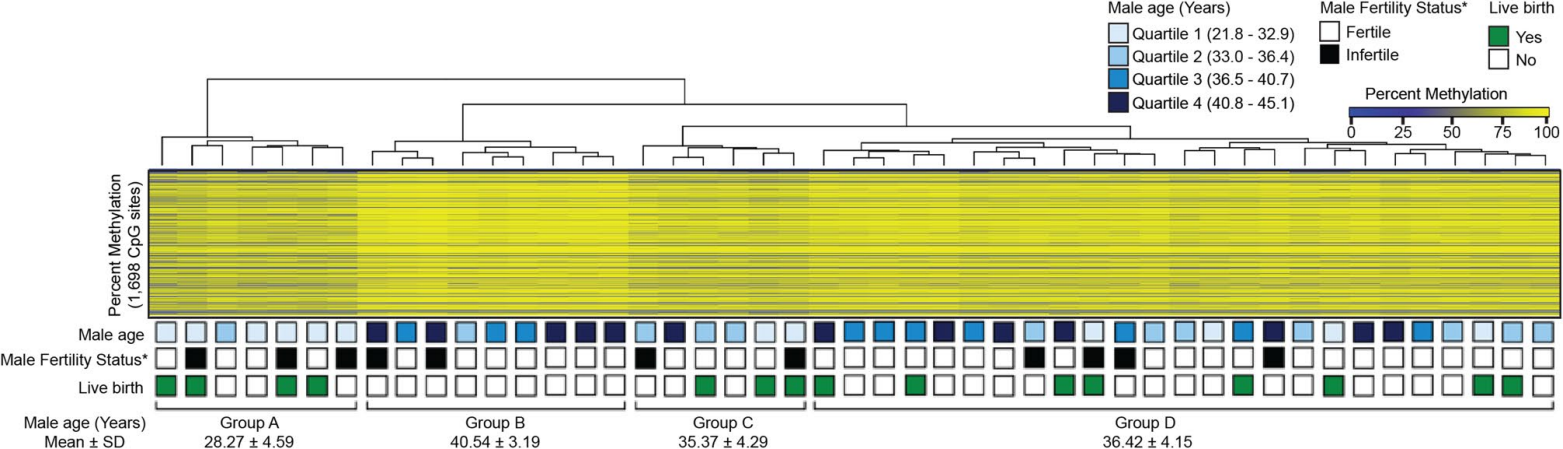
Unsupervised clustering of age-related sperm methylation at individual CpG sites (n = 1698). Boxes below the heatmap are color-coded representing individual-level data (n = 47) including quartiles of male age, male fertility status (fertile vs. infertile), and successful live birth (yes or no).

We next utilized unsupervised clustering of the 1698 significant individual sperm CpGs associated with male age to examine their relationships with the likelihood of fertilization and live birth (Fig. 6). Unsupervised clustering of age-associated methylome profiles resulted into four main groups with groups A and B having the youngest and oldest age (mean ± SD: 28.3 ± 4.6 years and 40.5 ± 3.2 years, respectively). The oldest group (group B) had distinct CpG hypermethylation profiles compared to the youngest age group while intermediate methylation was observed for groups C and D, suggesting a linear dose–response between male age and sperm methylation. When evaluating live birth in these groups defined by age-associated methylome profiles, 57% (n = 4 of 7) in the youngest group and 35% (n = 11 of 31) in the two intermediate age groups had successful live birth; however, no live births (n = 0 of 9) was observed in oldest group of men (group B). Interestingly, this group with no live births not only had the highest overall mean age, but also included younger men who had older sperm methylome profiles (Fig. 6).

### Sperm DNA methylation as a mediator of male age on ART outcomes

**Figure 7:**
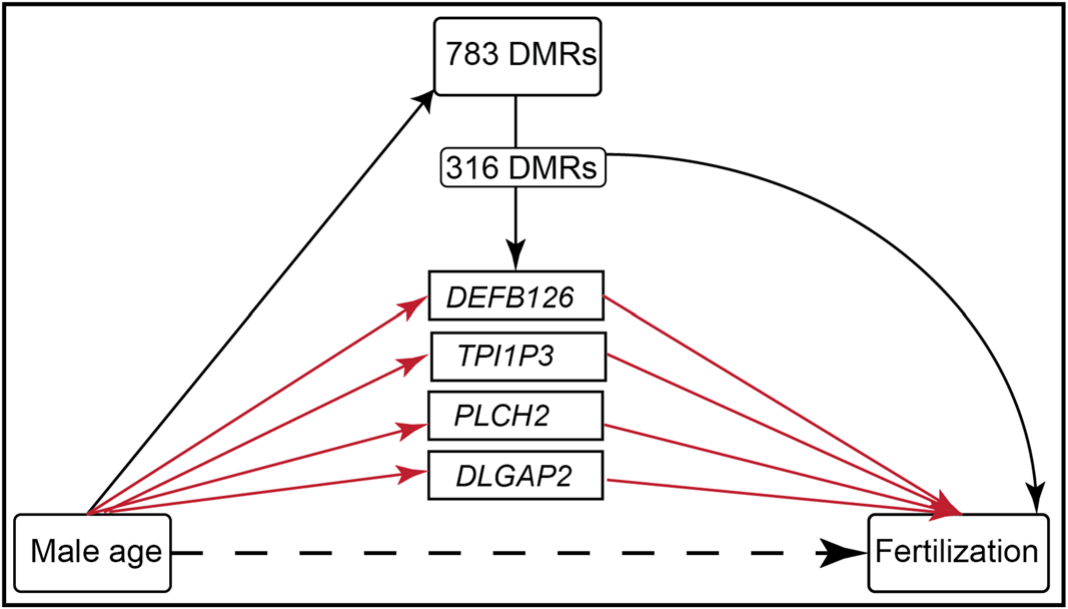
Schematic representation of mediation analyses for fertilization. The estimated association is provided on each pathway: the association between male age and each mediator; the association between each mediator and the outcome, and finally the remaining association of male age on the outcome, which is not mediated the DMRs (i.e. the direct effect). Total effects (TE) of age on outcomes is considered to be the sum of natural direct effect (dashed arrow) and natural indirect effect (red arrows).

We performed high dimensional mediation analyses to evaluate the role of sperm DNA methylation in male age-associated probability of fertilization and live birth, and to identify candidate DMRs as mediators. Among the 783 age-associated sperm DMRs within 1500 bp of the transcriptional start site of genes, 316 sperm DMRs were significantly associated (q < 0.05) with fertilization. Further screening by sure independence screening (SIS) identified four potential sperm DMR mediators: *DEFB126, TPI1P3, PLCH2, and DLGAP2* (Fig. 7 and Supplementary Table S7). The overall natural indirect effect (NIE) for the four DMR mediators is associated with lower odds of fertilization (NIE: 0.96, p < 0.05), suggesting that each 1 year higher male age was associated with approximately 4% lower odds of fertilization acting through sperm DNA methylation at these four DMRs. Additionally, the NIE of age on fertilization acting through these four sperm DMRs accounted for 63% (95% CI 41%, 86%; p < 0.05) of the total effect of male age on fertilization (OR = 0.92; 95% CI 0.89, 0.96).

A similar process was used to evaluate mediators of age effect on live birth as described previously for fertilization. High dimensional screening of all 783 age-associated sperm DMRs suggested three potential mediators consisting of four genes, *CHRNB1, FGF11, AC005017.2*, and *MYOF* (Supplementary Figure S1 and Table S7). The mediation analysis suggested that each 1 year higher male age was indirectly associated with lower odds of live birth, acting through methylation of these genes (NIE: 0.95, p < 0.05). The proportion of the OR representing the total effect of male age on live birth mediated by these DMRs was 81% (95% CI − 36.4%, 198%).

## Discussion

With secular trends toward delayed parenthood in developed countries, gamete quality is of clinical significance among couples or partners with advanced reproductive age. The utility of epigenetic aging based on measurements in somatic tissue has been highlighted in numerous studies^33,34^. In contrast, epigenetic aging in male germs cells on reproductive outcomes has been largely unaddressed to date. In this study, we show that male age is associated with extensive changes in sperm DNA methylation profiles, both at individual CpG sites and regional levels. Age-associated methylation was enriched at genes known for their role in early-life development and neurobehavior among others. To our knowledge, our results are the first to identify sperm DNA methylation as a likely pathway by which male age is associated with diminished reproduction outcomes in humans.

Spermatogonia are highly proliferative and self-renewing. Spermatogenesis commences at puberty and continues throughout adulthood, so that stochastic or induced changes in DNA methylation likely accumulate with age during the reproductive lifespan of the adult male. In regional analyses, sperm DMRs were associated with 784 genes that were significantly enriched in pathways important for biological functions such as embryonic, structural and central nervous system development, and cellular signaling. Of particular relevance are the findings that similar gene ontology (GO) terms were found among DMRs in CD4^+^ T cells comparing newborns and centenarians^39^ as well as in CD4^+^ cells comparing middle aged (40–55 years) and long-lived (> 90 years) individuals^40^. Within these broad GO categories, age-associated sperm DMRs were identified in several key genes that encode for transcription factors essential embryogenesis and neurodevelopment such as *WNT1, GATA4, ZIC1*, and *HOXB6*. Interestingly, age-associated methylation changes in mouse sperm were also enriched in genes important for Wnt/β-catenin signaling pathway^13^. Furthermore, while *ZIC1* encodes a transcription factor necessary for normal cerebellar development in human^41^, its mutation has been linked to cranial malformation and learning impairment in children^41^. Lastly, previous research has indicated that nucleosome-associated regions of sperm DNA, as compared to protamine-associated regions, may be important for embryonic development^42,43^. However, we found that age-associated sperm DMRs, contrary to our a priori hypothesis, were depleted, not enriched, in nucleosome regions, suggesting that sperm DNA methylation may mediate the influence of male age on reproductive outcomes independent of retained nucleosomes.

Advanced male age at conception has also been linked to an increase in risk in neurobehavioral issues in offspring. Interestingly, accumulating epidemiological evidence shows methylation signatures of neuro-developmental genes in sperm are particularly sensitive to advanced male age^23,44^. Similarly, in mice, age-associated sperm DNA methylation were transmitted to offspring with effect on both behavior and gene expression at regions known to be linked with schizophrenia and bipolar disorder^45^. In our analyses, we also identified several genes that were differentially methylated by male age including *DRD3, SYNGAP1, HTR2A*, and *GRIN1*. Hypermethylation of *DRD3*^46^ and *HTR2A*^47^ has been reported to be significantly associated with the risk of schizophrenia, while hypomethylation of *GRIN1* has been linked to depression in children^48^. Genetic alterations of *SYNGAP1* has also been linked to social dysfunctions and intellectual disability^49,50^. Together, these findings suggest that DNA methylation profiles of sperm from older fathers may contribute to the increase risk of abnormal embryo development and may play an important role in the neurodevelopment of offspring.

Numerous studies have attributed the association between higher male age and poorer reproductive outcomes to be driven by age-associated increases in sperm DNA damage^51^, chromosomal abnormalities^52^, as well as poor semen parameters^53,54^. Although some studies have reported no associations between sperm methylation and ART outcomes, they are limited by the use of global measures of methylation^55^ and/or limited to targeted approaches at imprinted and Y chromosome loci^56^. In contrast, genome-wide analyses showed that distinct sperm methylation profiles associated with embryo quality status among participants seeking infertility treatment^57^, suggesting that sperm methylation profiles could provide predictive value on subseqent embryo development. In our mediation analyses, we found that sperm DMRs in four genes, *DEFB126, PLCH2, TPI1P3* and *DLGAP2*, significantly mediated the relationship between male age and diminished fertilization. Of particular significance are the findings for *DEFB126*, which is a member of the beta-defensin protein family and is involved in several aspects of sperm function. DEFB126 is a main component of glycocalyx, which covers the entire surface of sperm and confers protection against the female immune system, enhances motility and facilitates binding to the zona pellucida^58–60^. Not surprisingly, loss of function of DEFB126 in males is associated with lower reproductive success. For example, men who are homozygous for the common *DEFB126* 2-nt frameshift deletion (*del/del*), have longer time-to-pregnancy among couples from the general population^61^ and lower clinical pregnancy rates among couples undergoing intrauterine insemination^62^. Additionally, sperm from *del/del* males exhibited an 84% reduction in penetration assays, a surrogate for cervical mucus, compared to other genotypes^61^. Similarly, a 4-nt frameshift deletion in *DEFB126* has also been associated with male infertility^63^. Most recently, co-culture of immotile testicular sperm with full-length DEFB126 protein compared to mutated isoforms resulted in increased sperm motility^64^. Our results suggest that the age-dependent increase in *DEFB126* methylation might also contribute to declines in reproductive success.

In addition, *DLGAP2* functions in synapse organization and signaling in neuronal cells and is implicated as a candidate gene for autism and schizophrenia^65^. Aberrant methylation of *DLGAP2* in sperm has been linked to male infertility^66^, as well as cannabis use in humans and THC exposure in rats^67^. Interestingly, rat offspring whose fathers were exposed to THC displayed differential methylation in the nucleus accumbens, suggesting an intergenerational inheritance of sperm methylation^67^. Despite the cell signaling function of *PLCH2*^68^, as well as the overexpression of the pseudogene, *TPI1P3*, in the testis, their direct involvement in mediating the effects of male age on fertilization is unknown.

Our high dimensional mediation analyses for male age on live birth also identified three sperm DMRs that were associated with four genes, *CHRNB1, FGF11, MYOF*, and *AC005017.2*. Of potential relevance, *FGF11* is a member of the fibroblast growth factor gene family, which is involved in a numerous biological functions such as embryonic and nervous system development^69,70^. *MYOF*, a member of the ferlin gene family, displays calcium mediated membrane functions for muscle functions in humans^71^; however, ferlin genes in other species such as *C. elegans* and Drosophila melanogaster are essential for reproductive success as mutations result in male sterility due to defects in fertilization^72,73^. *CHRNB1,* which shares a CpG island with *FGF11*, encodes the beta subunit of the muscle acetylcholine receptor; however, it is unknown how age-associated changes in sperm methylation of *CHRNB1* or the pseudogene, *AC005017.2,* influences the probability of live birth.

Age-associated alterations in DNA methylation are profound, providing the basis for use of statistical models to predict chronological age from methylation profiles in most somatic tissue^30,31^. The 1698 CpGs we identified with age-associated methylation in sperm are distinct from those used for chronological age prediction in somatic tissue, such that only 3 CpGs overlapped with Levine et al.’s 513 phenoage CpGs^33^ (Supplementary Table S8) while zero CpGs overlapped with Hannum’s 89 CpGs^31^ and Horvath’s 353 CpGs^30^. To date, one study by Jenkins et al. has examined the predictive ability of sperm methylation profiles on chronological age. Among 329 samples from infertile patients, sperm donors, and individuals from the general population, sperm methylation of 51 genomic regions via Illumina’s 450K that reproducibly predicted individual’s chronological age regardless of fertility status^35^. However, only 30% of these unique genomic regions used for these prediction models overlapped with our age-associated sperm DMRs (Supplementary Table S9). Possible explanations for the minimal overlap between age-associated sperm DMRs include differences in age distribution, such that our study only included men seeking IVF treatment and resulted in a narrower but clinically relevant age range of 22–45 years compared to Jenkins et al. with a male age range of 22–70 years and included men from the general population and those seeking fertility treatment. Additionally, technical variation in data processing, normalization, and statistical analysis approach (i.e., modeling m-values vs. beta-values for DNA methylation) could also account for differences in sperm DMRs. For example, 24 CpGs used in the Jenkins’ age prediction calculator were removed prior to our analyses in our multi-step data preprocessing pipeline due to potential cross-hybridization and localization near known SNPs. Not surprisingly, Jenkins’ age prediction calculator yielded a slightly attenuated correlation in our samples (actual vs predicted age R^2^ = 0.59; p < 0.001) compared with their 10 individual independent cohort validation (R^2^ = 0.77; p = 0.0016)^35^. Interestingly, when we stratified our results based on male infertility status or successful live birth, similar associations between actual and predicted age were found compared to Jenkins’ age prediction calculator (Supplementary Figure S2: infertile group; R^2^ = 0.75; p < 0.001 or no successful live birth; R^2^ = 0.77; p < 0.001). Lastly, it must be noted that the age-associated sperm DMRs in our study were slightly biased towards hypermethylation (57.0%), which is consistent with a recent study by Denomme et al.^44^ who also reported a slight bias toward hypermethylation (56.7%) of sperm DMRs. However, these findings contradict those of Jenkins et al. where only 5.5% of age-related sperm DMRs were hypermethylated^23^. The discrepancy of the direction of methylation change with age between these studies warrants further investigation in additional cohort studies.

Our study has several strengths. First, one unique feature of this study was the utilization of gradient centrifugation to isolate only motile fraction of the sperm, which represent the fraction with highest fertilization capacity. In a non-clinical natural pregnancy setting, sperm with higher motility have greater likelihood of fertilizing oocytes, which may help make these findings applicable to the general population. Additionally, our methylation analyses utilized the aliquots of the same semen samples that were used for ART procedures thus eliminating potential biases associated with obtaining independent semen samples. Moreover, use of gradient centrifugation represents standard clinical practice, so that these results have clinical relevance. Nevertheless, we recognize that there are some limitations to our study. Our novel findings are from a modest sample size of 47 couples. Additionally, participants include a mix of couples with male and/or female factor infertility seeking infertility treatment, and infertility status may play a complex role in the relationship between epigenetics and reproductive outcomes. To address this issue, we adjusted for infertility status and utilized stratified models in sensitivity analyses. Stratification by either male or female fertility status did not appreciably change the overall interpretation of our IVF outcome findings (Supplementary Table S1). We also recognize that assessment of DNA damage, such as DNA strand breaks, were not measured in our study and could possibly influence the effect of age on reproductive outcomes. Taken together, larger multi-center studies are warranted to validate and further refine our results with the inclusion of additional clinical information, such as hormone profiles and DNA damage, and to ensure generalizability across different infertility treatments.

## Conclusion

Through the use of genome-wide methylation analyses, our study is the first, to our knowledge, to identify methylation profiles that may serve as a potential mechanism by which male age negatively affects reproductive success (e.g., fertilization and live birth rates) in an ART setting. Such information may inform clinical care by identifying couples who have poor prognosis for fertilization success from infertility treatments such as timed intercourse, intrauterine insemination and traditional IVF compared to ICSI.

## Materials and methods

### Study population

The Sperm Environmental Epigenetics and Developments Study (SEEDS) is a prospective observational cohort study aimed at investigating the associations of male preconception endocrine disrupting chemical exposure with sperm epigenetics and subsequent early-life development. Participants were recruited from couples undergoing fertility treatment at Baystate Medical Center located in Springfield, Massachusetts. The inclusion criteria were male partners between 18 and 55 years old without vasectomy and fresh ejaculate sperm used for IVF treatment. Relevant demographics (race, age, height, and weight), lifestyle factors (current and past alcohol and cigarette use), and medical history (diagnoses of infertility) data were collected by clinic personnel during the IVF cycle for both partners. Male fertility diagnoses were based on semen quality parameters according to 2010 World Health Organization (WHO) reference values^74^ while female fertility diagnoses were based on CDC criteria such as polycystic ovarian syndrome, anovulation, tubal factors, diminished ovarian reserve, endometriosis and other factors^75^. Females received ovarian stimulation protocols based on infertility diagnoses and prior stimulation history, and egg retrieval was carried out about 36 h after the leading follicle reached 18–20 mm diameter as previously described^76^. The analytic sperm sample considered for the analyses described here comprised the first 48 men recruited for SEEDS with available data for sperm 450K methylation. One sample was removed from analyses based on assessment of outliers for DNA methylation. Written informed consent was obtained from eligible males who showed voluntary interest in participating in the study by the attending physicians. The study is approved by the institutional review boards at Baystate Medical Center and at the University of Massachusetts Amherst (IRB number: BH-12-190).

### Embryo quality assessment

Embryos were evaluated on a five-point scale (1 being the best and 5 being the worst quality) on days 3 and 5 post-insemination, during the cleavage stage and blastocyst stage, respectively, as previously described^77^. A separate category was reserved for all arrested embryos at both time points. At the cleavage stage, embryos were evaluated morphologically for the presence or absence of blastomere multinucleation, symmetry, cell number, and amount of fragmentation. At the blastocyst stage, embryos were evaluated for the developmental stage including the expansion of blastocoel and quality of trophectoderm and inner cell mass. At both time points, grades 1–2 were classified as high quality embryos, similar to that in our previously published study^77^, while at day 5, grades 1–4 were additionally classified as transferrable quality.

### Sperm collection and DNA isolation

Fresh semen samples were collected in a sterile plastic specimen cup after a recommended abstinence period of at least 48 h according to standard IVF protocol. To separate motile sperm from somatic cells as well as abnormal and non-motile sperm cells, semen samples were processed using a two-step gradient fractionation (40% and 80%). Sperm DNA from motile-enriched fraction were isolated according to our previously published protocol^78^ and used to generate sperm methylation data. As part of the routine protocol, trained embryologists microscopically examined all samples post-gradient fractionation, which were observed to be free of somatic cell contamination.

To further confirm findings were not influenced by somatic contamination, we analyzed methylation at imprinted genes, which have been published in a previous publication^28^. We showed that across identified 203 imprinted loci of 18 imprinted genes^79^, methylation levels of all maternal and paternal imprinted loci were < 10% or > 85% respectively, indicating negligible somatic cell contamination in the sperm samples. Here, we present methylation of maternal imprinted gene, *DLK1,* known to be differentially methylated between sperm and somatic tissues^35^ (Supplementary Figure S3). Average methylation for all participants was below 5% and male participants who were classified as infertile (shown in red) according to 2010 WHO cutoffs^74^ displayed no difference in average methylation at *DLK1* locus compared to fertile men (mean (SD): 2.87% (1.22) and 2.85% (1.06), respectively; p = 0.90). This data provides strong evidence that the reliability and validity of our results are not compromised by somatic contamination and participants’ fertility status.

### 450K analyses

Genomic sperm DNA (400 ng) was analyzed using Illumina 450K Infinium Methylation Beadchip, which allows a genome-wide coverage of over 450,000 methylation sites. Batch effects were minimized by randomizing samples within and across beadchip. The beadchip was run at the Applied Genomics Technology Center at Wayne State University. We performed within-array normalization and dye-bias equalization of Type I and Type II probes^80^ using normal-exponential convolution method (Noob)^81^ via minfi package in R^82^. Batch effects were corrected with the ComBat function in the sva package^83^ while cross-hybridizing probes and sex chromosome probes were removed using the DMRcate package^84^. Of the total 485,512 individual CpG sites interrogated for each participant, 75,164 sites were excluded after preprocessing, leaving a total of 410,348 CpG sites available for downstream analyses. The raw data can be found on GEO Accession Viewer (Accession Number GSE102970).

### Sequenom validation

Validation of our 450K data was performed using Sequenom mass array for both our significant and non-significant CpGs and the results are reported in our previous publication^28^. All show excellent agreement between the two platforms (e.g., cg14156792, Spearman r = 0.939, p < 2.2E−16; cg24997886, r = 0.911, p < 2.2E−16; cg19101893, r = 0.785, p = 6.25E−11). The two CpGs associated with male age (cg07462448 and cg04473763) displayed strong correlations (Spearman r = 0.89, p < 2.2E−16 and r = 0.77, p = 2.43E−10, respectively).

All methods were performed in accordance with the relevant guidelines and regulations.

### Statistical analyses

We first sought to determine the association between male age and ART outcomes: fertilization status, high quality embryos, and transferable quality status. Since we have repeated measurements of ART outcomes (multiple oocytes per couple), we modeled these outcomes as a proportion. The fertilization outcome is defined as the proportion of successful fertilization attempts for each couple; and similarly, for high quality embryo and transferable quality outcomes. This modeling approach makes it explicit that our unit of analysis is a couple and not an oocyte. We also included the number of oocytes per couple as observation weights in all logistic regression analyses performed involving fertilization, high quality embryos and transferable quality outcomes. The weighted logistic regression model accounts for the variability in the number of repeated measurements for each couple. All estimates represent the increase in the odds of the outcome associated with a 1-year higher male age, after adjusting for male body mass index (BMI), male infertility status based on 2010 WHO reference values^74^, male smoking and female age. These covariates were selected based on biological plausibility and after univariate screening with the outcome (p < 0.1). Statistical significance was defined as p-values < 0.05.

We then sought to determine the association between male age and sperm DNA methylation at individual CpG sites and regionally. To balance both validity and interpretability of our analyses, we conducted statistical analyses using both β-values and M-values, defined as the logit transformation of the β-values log (β/(1 − β)). To identify differences in percent methylation associated with male age, we used M-values due to its better adherence to homoscedasticity in linear models^85^. However, to facilitate the biological interpretation of our results, we reported effect estimates from models using beta-values, which provide CpG methylation values between 0 and 100%.

Associations between male age and sperm methylation were first analyzed at individual CpG sites using CpGassoc^86^. Next, regional analyses used A-clustering algorithm^87^ to identify co-regulated CpGs (≥ 2 correlated CpG sites) within 1000 base pairs. A total of 7771 co-regulated regions were identified and formed the unit of our regional methylation analyses. General estimated equations (GEE) were used to identify differential methylated regions (DMRs) by using a linear link and an exchangeable correlation to account for the correlated structure of the CpGs sites within a region^88^. All methylation analyses were adjusted for male covariates based on biological plausibility and included BMI, smoking status, and male infertility status. All p-values were adjusted for multiple testing via Benjamini-Hochberg (q < 0.05) and Bonferroni correction.

### Bioinformatics analyses

Prior to ontology analyses, each methylation region was assigned the closest gene within 1500 bp of TSS using GRCh37 assembly data from ENSEMBL via the annotatePeakInBatch function from the ChIPpeakAnno R package (version 3.6.5). We used metascape (http://metascape.org)^89^ to determine the enrichment of age-associated sperm DMRs with known biological pathways and regulation. We determined genic (enhancers, promoters, exons, introns, intergenic regions) and CpG features (island, shelves, shores, open sea) of methylated regions via annotations from Ensembl. For nucleosome retention vs. protamine designation of methylation regions, the publicly available dataset from Donkin et al. was used^38^. Binding site locations for transcription factors such as *EZH2* and *CTCF* were retrieved from ENCODE. Fisher’s exact test was used to test for significant enrichment or depletion of each feature in the statistically significant DMRs (GEE models; q < 0.05) versus the entire set of methylated regions.

### Mediation analysis

Analysis of mediation was conducted to evaluate sperm DNA methylation as a pathway through which male age might be related to ART outcomes. Analysis of the overall association of male age with these outcomes was described previously. Having identified the male age-associated DMRs, further screening was conducted to focus on those 783 DMRs that fall within 1500 bp of the TSS of the nearest gene, making them more likely to play a role in gene regulation. We used a two-stage screening procedure to reduce the number of DMRs to be evaluated in mediation analysis. In the first stage, we ran multiple logistic regressions of the outcome (as described previously) on each DMR adjusting for female age, male BMI, male smoking and male infertility status, and computed BH-adjusted p-values (q < 0.05) for the significance of each DMR. In the second stage, we used the R package Sure Independence Screening (SIS) followed by Minimum Concave Penalty (MCP) to further reduce the set of DMRs found significant in the first stage to be smaller than the sample size^90^. We performed a 'leave-one-out' approach where we iteratively removed one observation at a time from the data set and performed DMR screening using SIS; then we selected the DMRs that were identified by SIS in at least 20% of the iterations to be the final set of DMRs used in the mediation analyses. We then performed a regression-based multiple mediation analysis^91^ to assess mediation of the association between male age and ART outcomes (fertilization proportion and live birth status) by sperm methylation using this final set of DMRs. Analyses controlled for female age, male BMI, male smoking, and male infertility status. As described previously, analyses for the fertilization outcome included weights to account for the total number of oocytes per couple; however, weights were not used for models of live birth since only a single embryo was considered. For each outcome, we reported estimates and confidence intervals for the *natural indirect effect (NIE)*—defined as the effect of a 1 year increase in male age on odds of the outcome transmitted through the sperm DMR, and the *mediation proportion*—the proportion of the total effect of male age on odds of the outcome represented by the NIE indicating the association of male age with outcomes via sperm DNA methylation.

We note that our mediation analysis utilized male age and DMR values that were on continuous scales while fertilization and live birth outcomes were modeled on logistic scales. We followed the adjustment procedure proposed^92^. This approach entails computing a rescaling factor to correct for this incompatibility for the sake of validity of statistical inference and interpretation of the natural indirect effect and proportion mediated.

## Supplementary Information

Supplementary Information.
